# Warm Needling Therapy and Acupuncture at Meridian-Sinew Sites Based on the Meridian-Sinew Theory: Hemiplegic Shoulder Pain

**DOI:** 10.1155/2015/694973

**Published:** 2015-10-01

**Authors:** Hong Zhao, Wenbin Nie, Yuxiu Sun, Sinuo Li, Su Yang, Fanying Meng, Liping Zhang, Fang Wang, Shixi Huang

**Affiliations:** ^1^Institute of Acupuncture and Moxibustion, China Academy of Chinese Medical Sciences, No. 16 Dong Zhi Men Nei Nan Xiao Jie, Dongcheng District, Beijing 100700, China; ^2^Guang'anmen Hospital, China Academy of Chinese Medical Sciences, Beijing 100053, China; ^3^Mentougou Hospital of TCM, No. 3 Xinqiao South Street, Mentougou District, Beijing 102300, China

## Abstract

This study was performed to evaluate the effectiveness and safety of warm needling acupuncture at meridian-sinew sites based on the meridian-sinew theory in the treatment of hemiplegic shoulder pain (HSP) after stroke. In total, 124 subjects were randomized into a treatment group and control group. In the treatment group, warm needling therapy and acupuncture at meridian-sinew sites based on the meridian-sinew theory were performed. In the control group, usual care therapy was applied. The visual analog scale (VAS) score, range of motion (ROM), and Barthel index (BI) were used to evaluate treatment effectiveness. At 2 weeks of treatment, the VAS score, ROM, and BI had obviously changed from baseline in the two groups (*P* < 0.01). The changes in the VAS score and ROM in the treatment group were significantly greater than those in the control group (*P* < 0.01). At the 3-month follow-up after treatment, the changes in the treatment group were significantly greater than those in the control group (*P* < 0.01). This study indicates that warm needling therapy with acupuncture at meridian-sinew sites based on the meridian-sinew theory is effective for HSP.

## 1. Introduction

Hemiplegic shoulder pain (HSP) is one of the most common complications after stroke, affecting 16–84% of impaired stroke survivors [1, 2]. HSP usually occurs in the early stage after stroke but may also develop in the chronic stage. HSP may limit activity of the shoulder joint, which impacts the limb motor function, disturbs the rehabilitation process, extends the length of hospitalization, and affects the patient's quality of life [3, 4].

The causative factors of HSP are unknown but are generally considered to involve conditions such as muscular hypertonia, subluxation of shoulder joint, and thalamus damage. HSP is currently treated with physiotherapy, massage therapy, strapping, slings, intra-articular or subacromial corticosteroid injections, suprascapular nerve blocks, percutaneous or superficial electrical muscle stimulation, intramuscular botulinum toxin type A injections, or other supportive therapies to minimize glenohumeral subluxation [5, 6]. However, challenges remain because there is no specific evidence for most of these treatments [7].

The efficacy of acupuncture for shoulder pain has been verified [8]. A review on the effectiveness of acupuncture therapy for shoulder pain after stroke showed that acupuncture combined with exercise is effective for this condition [9]. HSP clinically manifests as a frozen shoulder or adhesive capsulitis, which is consistent with the manifestations of meridian-sinew disorder in traditional Chinese medicine (TCM). Meridian-sinew therapy is a component of TCM theory used by ancient TCM masters in which the 12 routes of motoricity are used to elaborate the physiological and pathological laws of syndesmology, mycology, and the attached tissues in the human body [10]. An early preliminary experiment indicated that based on the meridian-sinew theory warm acupuncture therapy can relieve pain and improve range of motion (ROM) of the shoulder in patients with HSP after stroke [11]. However, there is currently no evidence for the effectiveness of acupuncture based on the meridian-sinew theory for treatment of HSP. Therefore, this study was designed to evaluate the efficacy and safety of acupuncture based on the meridian-sinew theory and moxibustion for the treatment of HSP.

## 2. Subjects and Methods

### 2.1. Design

This was a prospective, simple-blind, randomized controlled clinical study performed at Guang'anmen Hospital of the China Academy of Chinese Medicine Sciences and Mentougou Hospital of TCM from January 2010 to March 2012. The subjects were randomized into two groups at a 1 : 1 ratio and SPSS was used. An independent physician who did not participate in the treatment and efficacy evaluation was permitted to disclose the envelopes according to the number sequence, and the patients were grouped based on the enclosed allocation plan.

At the first visit, the independent physician selected patients who met the inclusion criteria and randomized them into the two above-mentioned groups. Patients in the treatment group underwent acupuncture based on the meridian-sinew theory combined with moxibustion in five treatments a week. The control group underwent usual care (UC) therapy only. Treatment was performed for a total of 2 weeks, and follow-up was performed 3 months after the end of treatment.

### 2.2. Subjects

The patients were recruited through an advertisement in the registration hall and wards of the two above-mentioned hospitals: Guang'anmen Hospital of the China Academy of Chinese Medical Sciences and Mentougou Hospital of TCM. Informed consent was required before enrollment according to the clinical protocol. Recruitment was performed from January 1, 2010, to March 31, 2012. A total of 124 patients were recruited.

The inclusion criteria were as follows: (1) cerebral infarction or cerebral hemorrhage (confirmed by head computed tomography or magnetic resonance imaging), hemiplegia, paralysis, or quadriplegia; (2) pain and discomfort of the shoulder occurring at rest and/or movement after hemiplegia; (3) age of 18–70 years; (4) ipsilateral limb muscle tension with an Ashworth score of 1 or higher; (5) discontinuation of all analgesics and medications that may impact muscle tension 1 week before treatment; and (6) providing written informed consent and volunteering to participate in the study.

The exclusion criteria were as follows: (1) subarachnoid hemorrhage or transient cerebral ischemic attacks; (2) shoulder pain other than HSP; (3) serious primary diseases of the cardiovascular, liver, kidney, or hematopoietic system; (4) intolerance to acupuncture therapy; and (5) no desire to participate in the study.

### 2.3. Intervention

In the treatment group, acupuncture and moxibustion were applied to the meridian-sinew sites. The materials used in the treatment were acupuncture needles, sterile silver-handle prepacked needles (Hua Tuo single-use acupuncture needles; Suzhou Medical Co., Ltd., Jiangsu, China) without guide tubes (0.25 × 25 mm, 0.25 × 40 mm, or 0.25 × 50 mm), and a 1.5 cm long moxa stick (Han Yi brand; Wo-long Han Yi Moxibustion Factory, Nan Yang, China).

The meridian-sinew sites (stimulating sites) were detected by holding the distal end of the affected upper limb and assisting the patients to perform elevation, abduction, external rotation, internal rotation, and other movements while pressing the cord-like painful sites (meridian-sinew sites) along the pathways of the meridians of the limb.

The most common meridian-sinew sites used in this study were as follows:

 Jingjianci (sub-GB21): on the root of the neck, directly above the medial-superior scapular angle, on the junction of the upper bundle of the trapezius and levator scapulae.
 Jianyuci (sub-LI15): on the shoulder, at the terminal end of the anterior bundle of the deltoid, lateral to the clavicle and anterior to the acromion.
 Binaoci (sub-LI14): on the radial side of the humerus, inferior to the deltoid, on the anterior border of the lateral end of the triceps brachii muscle.
 Jianzhenci (sub-SI9): on the junction of the major and minor muscle and long head of the triceps brachii muscle on the back of the axilla.
 Jianliaoci (sub-TE14): on the back of the shoulder, at the terminal end of the scapular spine, posterior to the deltoid.
 Tianfuci (sub-LU3): on the shoulder, at the ridge of the greater tuberosity and lesser tubercle of the humerus.
 Jujianci (sub-Jujian): in front of the axilla, in the tendon of the subscapular muscle.
 Shousanlici (sub-LI10): on the radial side of the forearm, at the junction between the digital extensor muscle and supinator muscle.
 Yangchici (sub-TE4): the midpoint of the transverse crease on the dorsum of the wrist.

The treatment technique was performed as follows. The needles were inserted into the meridian-sinews for* Deqi*, and lifting, thrusting, and twisting were performed for 2 min. Next, two sites were selected and stimulated with the warm needling technique, in which the ignited moxa stick was attached to the handle of the needle and replaced once until it burned out. The needles were removed at the end of moxibustion therapy.

One treatment was administered for a maximum of 30–40 min five times per week for 2 weeks. Acupuncture and moxibustion were performed by physicians with more than 5 years of clinical experience in acupuncture and moxibustion.

The patients in the control group underwent UC therapy in the hospital five times per week for 2 weeks. Physical therapy (PT) is common in UC but has been recognized as an effective approach for the treatment of HSP [12, 13]. PT was administered by physical therapists who were blinded to the group divisions and had professional qualifications. The PT program was in accordance with the recommendations of officially published guidelines, including correct positioning of the joints, proper joint movement ranges and strengthening exercises, and performance of occupational therapy [14].

### 2.4. Outcomes

#### 2.4.1. Primary Outcomes

The primary outcomes were the visual analog scale (VAS) score and ROM.

The VAS is used to evaluate pain. It is a measurement instrument constituting a 10 cm line in which 0 represents “no pain” and 10 indicates “the worst pain imaginable.” The patient marks his or her subjective feeling of pain on the line. In the present study, the baseline pain level was taken to be the worst pain felt during a specific movement of the shoulder joint.

A 180° common protractor was used to measure the ROM of the shoulder, including the ranges of abduction, anteflexion, backward extension, and adduction.

#### 2.4.2. Secondary Outcomes

The Barthel index (BI) is used to evaluate the activity of daily living (ADL). The score is given in terms of the following variables: feeding, bathing, grooming, dressing, defecation, urination, toilet use, transfers (bed to chair and back), mobility on level surfaces, and climbing stairs. The highest possible score is 100 points.

The VAS score was evaluated after each treatment, and the other three scales (ROM, BI, and modified Ashworth score) were applied before and 2 weeks after treatment. The VAS score and BI were also determined 3 months after the end of treatment.

### 2.5. Follow-Up Therapies

Because of the different functional disturbances in most stroke survivors and for ethical reasons, patients were allowed to undergo other therapies during the follow-up period after the end of treatment, including rehabilitation therapy (such as PT and occupational therapy) and medication to relieve spasticity. However, rehabilitation therapies that might act directly on the hemiplegic shoulder and medication to treat central paroxysmal pain were prohibited.

### 2.6. Sample Size Calculation

Changes in the VAS scores before and after treatment were taken as the main effect indicators. According to the results of preliminary experiments, the VAS scores in patients with HSP who underwent warm needling acupuncture at meridian-sinew sites and UC treatment were reduced 5.5 ± 1.925 [11] and 4.06 ± 25.4 [15], respectively. Based on a noninferiority test with 80% power, an alpha level of 0.05, and a 20% dropout rate, the sample size was calculated as 62 patients in each group.

### 2.7. Statistical Analysis

Statistical analysis was performed with SPSS software (version 12.0; SPSS, Inc., Chicago, IL, USA). A two-sided test was adopted for the statistical analysis, and *P* ≤ 0.05 indicated a statistically significant difference. The independent *t*-test was applied for comparison of the measurement data between the two groups. The paired *t*-test was used for comparison of the measurement data within a single group.

## 3. Results

### 3.1. Trial Profile

From January 2010 to March 2012, a total of 167 patients with HSP visited the Mentougou Hospital of TCM and Guang'anmen Hospital of the China Academy of Chinese Medical Sciences. Of these patients, 43 were excluded for the following reasons: failure to meet the inclusion criteria (*n* = 21); having taken analgesics for other painful disorders (*n* = 8); the presence of cognitive impairment (*n* = 6); the presence of aphasia (*n* = 5); and refusal to participate in regular treatments five times per week (*n* = 3). Of the remaining 124 patients, 84 had not been treated with acupuncture in the past, and 40 had received acupuncture treatment 1 week previously.

**Figure 1:**
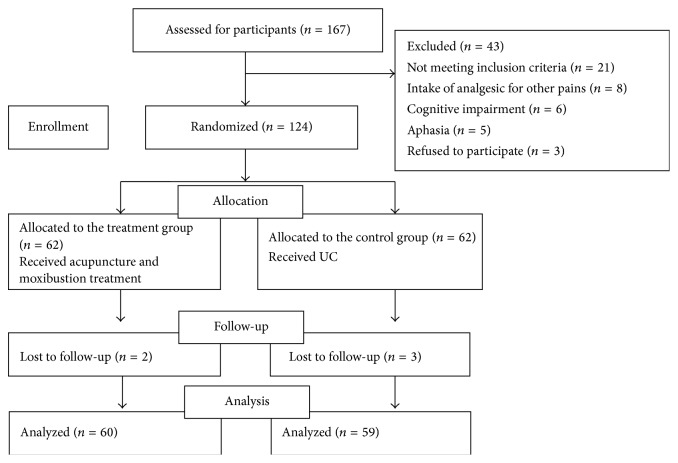
Participant flow diagram.

During the treatment, one patient in the treatment group discontinued the treatment because of sudden recurrence of cerebral infarction with aphasia happening, and one patient in the control group discontinued the treatment because of failure of subjective pain release. Both patients had completed more than 1 week of treatment; therefore, their evaluation data on final efficacy were transferred for the statistical analysis. During the follow-up, two patients in the treatment group were lost owing to recurrence of cerebral infarction and two in the control group were lost because of serious pulmonary infection and an incorrect phone number, respectively. The trial profile was presented in Figure 1.

### 3.2. Participants' Baseline Characteristics

Baseline characteristics of the patients in the two groups were showed in Table 1 with no significant difference.

### 3.3. Changes in VAS

**Figure 2:**
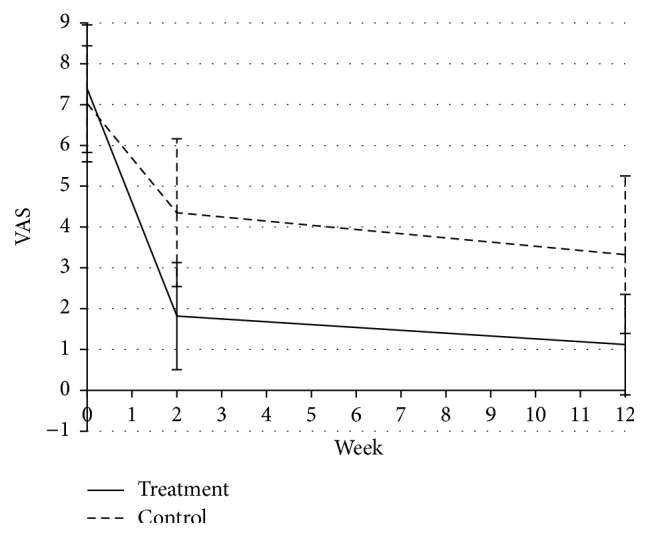
VAS scores in the two groups throughout the study.

There was no difference in the baseline VAS scores between the two groups. After 2 weeks of treatment, the VAS scores were significantly decreased from 7.39 ± 1.56 to 1.82 ± 1.31 in the treatment group (*P* < 0.001) but decreased from 7.02 ± 1.42 to 4.35 ± 1.81 in the control group (*P* < 0.001). The reduction was significantly different between the two groups (*P* < 0.01). During the 3-month follow-up period, the VAS scores decreased to 1.12 ± 1.23 (*P* < 0.001) but decreased to 3.32 ± 1.93 in the control group (*P* < 0.001). The reduction was significantly different between the two groups (*P* < 0.01) (Figure 2 and Table 2).

### 3.4. Changes in Shoulder ROM

**Figure 3:**
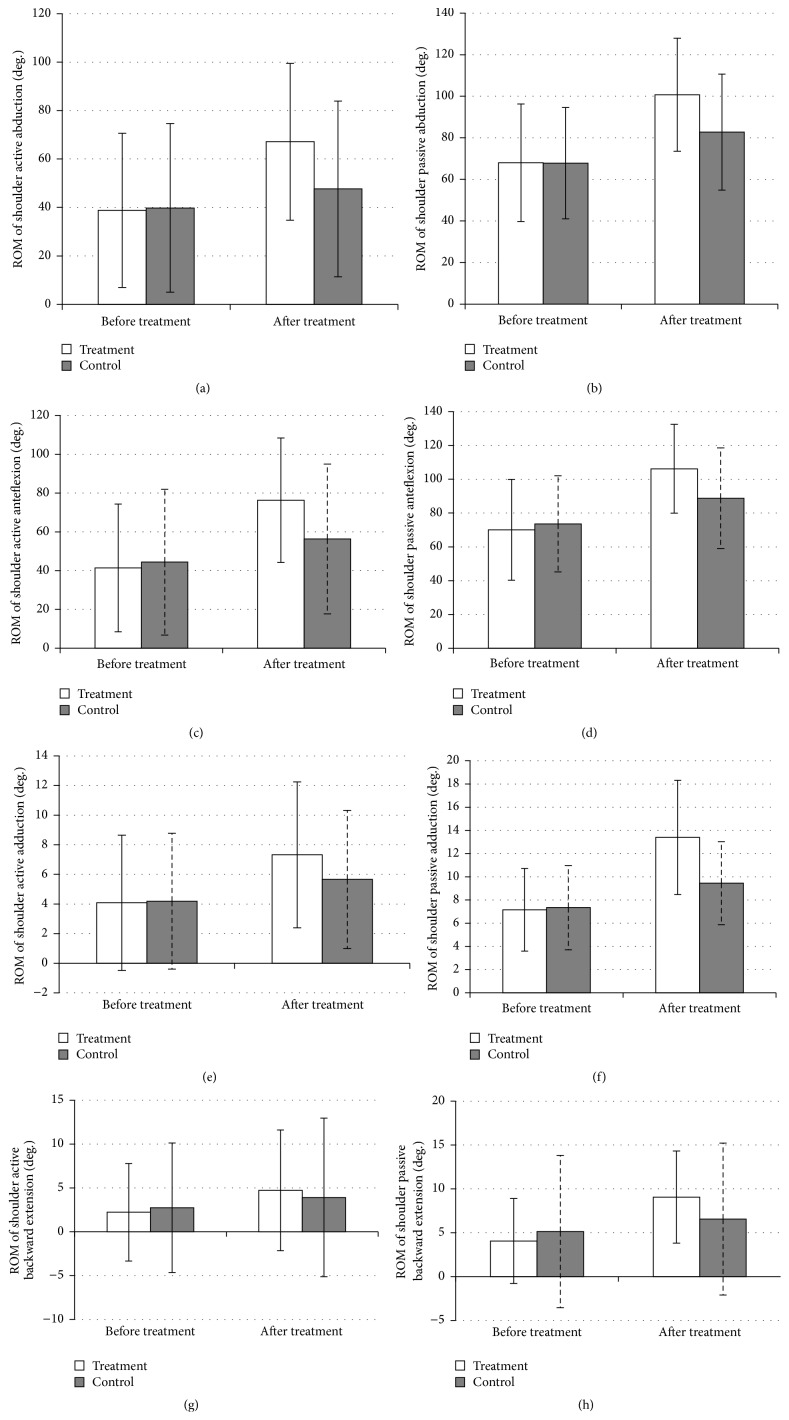
ROM of shoulder before and after treatment in the two groups.

There was no difference in baseline shoulder ROM in different directions between the two groups. After 2 weeks of treatment, both the active and passive ROM of shoulder abduction were greater than those before treatment in both groups (*P* < 0.01). There were significant differences in active and passive ROM between the two groups; the improvement in the treatment group was greater than that in the control group (both *P* < 0.01) (Figures 3(a) and 3(b)).

The ROM of active and passive anteflexion was greater than that before treatment in both groups (*P* < 0.01). There were significant differences in the active and passive ROM changes between the two groups; the improvement in the treatment group was greater than that in the control group (both *P* < 0.01) (Figures 3(c) and 3(d)).

The ROM of active and passive adduction was greater than that before treatment in both groups (*P* < 0.01). There were significant differences in active and passive ROM changes between the two groups; the improvement in the treatment group was greater than that in the control group (both *P* < 0.01) (Figures 3(e) and 3(f)).

The ROM of active backward extension was greater than that before treatment in both groups (*P* < 0.01). The increased ROM of backward extension in the treatment group was significantly greater than that in the control group (*P* < 0.01). The ROM of passive backward extension was greater than that before treatment in both groups. It was increased by 5.02 ± 2.74 degrees in the treatment group and 1.42 ± 3.50 degrees in the control group (*P* < 0.01). The increased ROM of backward extension in the treatment group was significantly greater than that in the control group (*P* < 0.01) (Figures 3(g) and 3(h)).

### 3.5. BI in the Two Groups

**Figure 4:**
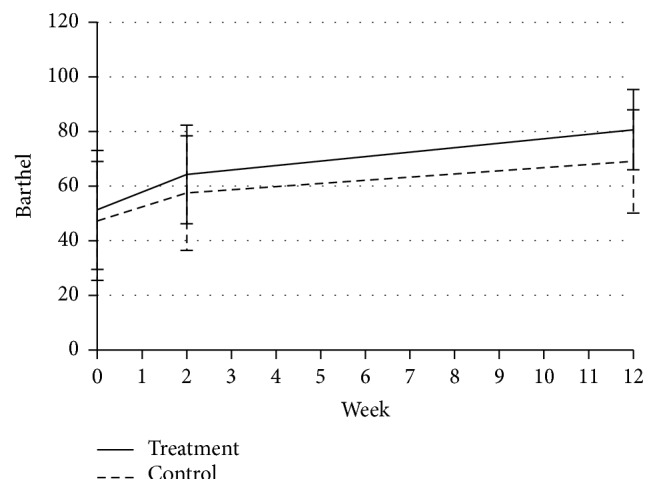
BI scores in each group throughout the study.

There was no difference in the baseline BI scores between the two groups. After 2 weeks of treatment, the BI scores were significantly increased from 51.29 ± 21.77 to 64.26 ± 17.99 in the treatment group (*P* < 0.001) but increased from 47.28 ± 21.74 to 57.45 ± 20.97 in the control group (*P* < 0.001). The improvement was not significantly different between the two groups (*P* = 0.25). At the 3-month follow-up after treatment, the BI scores increased to 80.65 ± 14.67 (*P* < 0.001) but increased to 69.03 ± 18.90 in the control group (*P* < 0.001). The improvement was significantly different between the two groups (*P* < 0.01) (Figure 4).

### 3.6. Safety

During the 2-week treatment, two patients in the treatment group (3.2%) reported pain. No other serious adverse events were reported.

## 4. Discussion

### 4.1. Key Findings

This study was a randomized controlled trial with a good design. We evaluated the effect of warm needling acupuncture at meridian-sinew sites based on the meridian-sinew theory on the treatment of HSP after stroke.

The study results showed that in light of the changes in VAS scores and ROM this therapeutic method not only alleviated pain, but also improved the motor function of the affected shoulder joint. The treatment group experienced a significant reduction in VAS scores during the 2 weeks of treatment and the 3-month follow-up period (*P* < 0.01). The results indicated the sustainable effect of this therapy. These beneficial effects not only were displayed at 2 weeks of treatment, but were also sustainable until the end of the study. For the patients who underwent UC therapy, improvements in shoulder pain and ROM were also achieved at both 2 weeks after treatment and at the end of study. However, there were statistically significant differences between UC and acupuncture therapy. The effects of pain alleviation and ROM improvement of the shoulder were much more obvious in the acupuncture and moxibustion treatment group.

In terms of the BI, ADL was better 2 weeks after treatment than before treatment in both the treatment and control groups. However, the changes were not significantly different from the baseline values (*P* = 0.25). At the end of study, the change in the BI was significant between the two groups (*P* < 0.01). The results showed that, compared with UC therapy, warm needling acupuncture therapy at meridian-sinew sites improved the ADL of the subjects and demonstrated long-term efficacy.

This study showed that acupuncture combined with moxibustion based on the meridian-sinew theory is a safe therapy for the treatment of HSP. Only two patients (3.2%) reported pain in the treatment group, and no other adverse events were observed.

### 4.2. Explanations

The meridian-sinew theory is an important component of acupuncture. Previous studies have shown that this theory has been used to relieve knee joint pain in the treatment of knee osteoarthritis [15]. However, clinical studies on the meridian-sinew theory and relevant therapeutic methods are rare.

**Figure 5:**
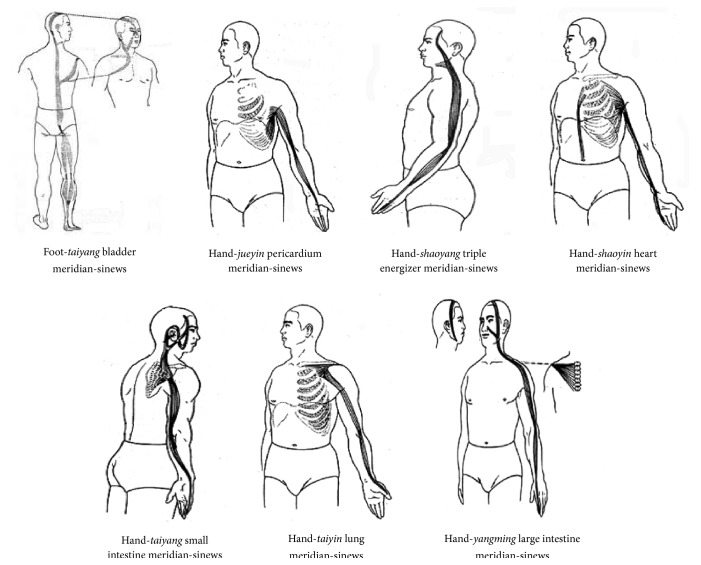
Running courses of seven meridian-sinews.

According to the ancient text* Huangdi Neijing* (*Yellow Emperor's Internal Classic*), seven meridian-sinews run along the upper extremity and shoulder joint, including the gallbladder meridian-sinew of foot-*taiyang*, the small intestine meridian-sinew of hand-*taiyang*, the triple energizer meridian-sinew of hand-*shaoyang*, the large intestine meridian-sinew of hand-*yangming*, the lung meridian-sinew of hand-*taiyin*, the heart meridian-sinew of hand-*shaoyin*, and the pericardium meridian-sinew of hand-*jueyin* [16]. The above-mentioned meridian-sinews run on the posterior, superior, anterior, inferior, and lateral of the shoulder joint separately (Figure 5). Cord-like or lumpy reactive sites are commonly detected in local areas of pain in patients with HSP after stroke in clinical practice. All of these reactive sites are located along the course of the seven meridian-sinews, and these sites match the locations of the meridian-sinew sites in the acupuncture theory. Hence, based on the meridian-sinew theory, the warm acupuncture theory was applied to the local meridian-sinew sites in the treatment of HSP in the present study. This treatment relieved shoulder spasticity and pain and improved the shoulder ROM.

## 5. Conclusion

On the basis of UC, warm needling acupuncture therapy at meridian-sinew sites relieves pain, improves ROM and ADL, and has no side effects in the treatment of HSP after stroke. This therapy also has a certain sustainable effect. In conclusion, warm needling acupuncture therapy at meridian-sinew sites is suitable for the treatment of HSP after stroke.

## Figures and Tables

**Table 1:** Baseline characteristics of the patients in the two groups.

Characteristics	Treatment group	Control group	*P* value
Age (years)^*∗∗*^	63.73 ± 11.65	65.53 ± 10.67	0.37
Sex (male : female)^*∗*^	44 : 18	45 : 17	0.82
Duration of stroke (days)^*∗∗*^	120.0 ± 224.232	104.5 ± 159.717	0.658
Duration of shoulder pain (days)^*∗∗*^	99.613 ± 208.545	74.194 ± 130.931	0.418
NIHSS^Δ^	5.98 ± 2.37	6.35 ± 2.44	0.40
Stroke type (infarction : hemorrhage)^*∗*^	51 : 11	53 : 9	0.87
Muscle strength (0 : I : II : III : IV)^*∗*^	8 : 1 : 15 : 25 : 13	9 : 2 : 12 : 25 : 14	0.95
Other treatments for shoulder pain			
Acupuncture and moxibustion^*∗*^	42	42	1.000
West treatment^*∗*^	62	62	1.000
Physical treatment^*∗*^	2	3	1.000

^Δ^NIHSS: National Institute of Health Stroke Scale.

^*∗*^Number presented for each group.

^*∗∗*^Mean ± SD presented for each group.

**Table 2:** Comparison of VAS scores in the two groups throughout the study.

	Treatment group	Control group	*P* value
	Mean ± SD	Mean ± SD	
Baseline	7.39 ± 1.56	7.02 ± 1.42	
After treatment	1.82 ± 1.31^*∗*^	4.35 ± 1.81	
Follow-up	1.12 ± 1.23^*∗*^	3.32 ± 1.93	
Changes from baseline to after treatment	5.56 ± 1.85	2.66 ± 1.61	0.00^*∗∗*^
Changes from baseline to follow-up	6.27 ± 1.75	3.36 ± 1.93	0.00^*∗∗*^

^*∗*^ *P* < 0.01 comparison of changes from baseline to after treatment and follow-up in each group.

^*∗∗*^ *P* < 0.01 comparison of changes from baseline to after treatment and follow-up between the two groups.
